# Tailoring communications to the evolving needs of patients throughout the cancer care trajectory: a qualitative exploration with breast cancer patients

**DOI:** 10.1186/s12905-016-0347-x

**Published:** 2016-10-18

**Authors:** Eun-Jung Shim, Jee Eun Park, Myungsun Yi, Dooyoung Jung, Kwang-Min Lee, Bong-Jin Hahm

**Affiliations:** 1 Department of Psychology, Pusan National University, Busan, Korea; 2 Department of Neuropsychiatry, Seoul National University Hospital, 101 Daehak-ro, Jongno-gu, Seoul, 03080 Korea; 3 College of Nursing, Research Institute of Nursing Science, Seoul National University, Seoul, Korea; 4 Department of Human Factors Engineering, Ulsan National Institute of Science and Technology, Ulsan, Korea; 5 Department of Psychiatry and Behavioral Sciences, Seoul National University College of Medicine, 101 Daehak-ro, Jongno-gu, Seoul, 03080 Korea; 6 Integrated Cancer Care Center, Seoul National University Cancer Hospital, 101 Daehak-ro, Jongno-gu, Seoul, 03080 Korea

**Keywords:** Breast cancer, Consultation timing, Doctor-patient communication, Doctor-patient empathy

## Abstract

**Background:**

Doctor-patient communication is a crucial aspect of patient care. This study explored the communication experience of patients in a cancer consultation over the course of the cancer continuum.

**Methods:**

In-depth interviews with seven breast cancer patients were carried out.

**Results:**

Themes related to communication experiences across the five phases of cancer consultation, from diagnosis to recurrence, were identified. The most salient issue is that patients also perceived cancer as ‘a disease of the mind’, which is not adequately cared for in consultation. This highlights the notion that cancer care providers should provide appropriate care for the psychological dimensions of the cancer experience with an empathic and sincere attitude during consultations. To this end, non-verbal aspects of communication that convey caring, support, and respect seem important. Furthermore, patients perceived that the consultation time was far shorter then they needed and reported that they felt pressured for time. Moreover, the stance taken by patients and the needs and preferences of patients varied across the phases of the cancer trajectory. As patients progressed through the phases of their treatment, they assumed more active roles in the course of their care and the need for more detailed information and questioning increased. Thus, ensuring that patients have opportunities to ask questions in the consultation is important.

**Conclusion:**

Current findings suggest that the efficacy of communication varies depending on which phase patients are in and that effective communication should be tailored to these evolving needs and preferences of breast cancer patients. Also, patients perceived that the consultation did not adequately address their need for information related to their care or their emotional issues associated with the cancer experience. It is therefore important to address their needs by paying particular attention to non-verbal aspects of communication that convey empathy and respect toward patients, as well as allowing patients to ask questions.

**Electronic supplementary material:**

The online version of this article (doi:10.1186/s12905-016-0347-x) contains supplementary material, which is available to authorized users.

## Background

Doctor-patient communication is a crucial aspect of patient care. It is associated with patient adherence [1] and increased use of alternative and/or unnecessary treatment [2]. It is also related to the satisfaction with care [3] and quality of life [4].

However, previous findings suggest that there are still unmet needs of cancer patients relating to the communication in consultations. For instance, breast cancer patients had high unmet information needs regarding their health and were dissatisfied with the information provided by care providers [5, 6]. Moreover, patients’ affective responses were not adequately addressed in medical consultations [7]. Although quality of life issues are discussed in cancer consultations, the focus is mainly on treatments and symptoms and other aspects, such as psychological health and social and spiritual dimensions of quality of life, are rarely addressed in cancer consultations [8]. Given the current state, it is not surprising that issues related to information and psychosocial aspects of cancer care are ranked highly among the most unmet needs in studies with cancer patients [9].

Furthermore, as patients might face different challenges and difficulties across the phases of the cancer continuum [10, 11], their needs and preferences might vary, including those regarding the communication, and the way doctors communicate with them should be responsive to these evolving needs. For instance, Dowsett and colleagues [12] examined preferences for communication style in cancer consultations with breast cancer patients and their relatives, and found that a preference for a patient-centered approach was especially prominent in the case of a poor prognosis. Also, a study by Vogel, Bengel and Helmes [13] with 135 German breast cancer patients found that information needs and decision making preferences can change during the course of treatment. Patients rated the quality of information received from physicians significantly better at baseline compared to 6 months later and shared decision making was uncommon in the first 6 months of treatment.

Although previous reviews suggest evolving needs and preferences relating to the communication in cancer consultations [5, 14], there have been relatively few studies conducted on the subject. To our knowledge, the study by Thorne and colleagues [15] is the first study that qualitatively explored communication needs in cancer care. According to Thorne et al. [15], what makes communication helpful in one phase of cancer care might not necessarily be helpful in another phases of cancer care, and it is important to explore the optimal ways of communication that effectively address these evolving needs and preferences. One appropriate strategy for exploring this topic is to ask the stakeholder, that is the cancer patients, directly. According to Wright et al. [16], patient perspective regarding effective communication should be taken into consideration when developing communication programs for care providers in oncology.

Despite its importance, studies that explore the communication experiences of cancer patients from their perspective, and throughout the continuum of cancer care, are few. In an attempt to understand effective communication strategies responsive to the evolving needs and preferences of breast cancer patients in cancer consultations, this study explores the communication experience in cancer consultations, over the course of the cancer continuum, using in-depth interviews with seven breast cancer patients.

## Methods

### Participants and procedures

Participants consisted of patients enrolled in a group meditation program at a university hospital psycho-oncology clinic. Inclusion criteria consisted of being over 20 years old and diagnosed and treated for breast cancer. Seven patients gave their informed consent and their characteristics are shown in Table 1.

**Table 1:** *Participant Characteristics* (*N* = 7)

	A	B	C	D	E	F	G
Age	44	56	44	54	50	38	35
Sex	F	F	F	F	F	F	F
Stage	I	I	II	II	II	III	II
Tx period	7M	1Y	3Y	9M	9M	9M	9M
Op	Y	Y	Y	Y	Y	Y	Y
CTx	Y	Y	Y	Y	Y	Y	Y
RTx	Y	N	Y	N	Y	Y	Y
Recurrence	N	N	Y	N	N	N	N
Current Tx	Y	N	Y	N	N	N	N

*Tx* treatment, *Op* operation, *CTx* chemotherapy, *RTx* radiation therapy

In-depth and semi-structured interviews began with a ‘grand-tour’ question: “Tell me about your communication experience from when you were in consultations with doctors as a cancer patient”. They were then given questions specifically addressing their experience throughout each phase of the cancer care trajectory, focusing on patients’ experiences of communication with doctors in consultations (Additional file 1). Interviews were recorded and fully transcribed verbatim.

This study was approved by the Institutional Review of Board of Seoul National University Hospital.

### Analytic strategies

Transcripts were analyzed using a content analysis approach. Transcripts were coded independently by team members and then examined by a ‘set team’. After coding and extraction of themes, a research team discussed the findings in order to foster multiple perspectives. When a disagreement occurred regarding the meaning of data or coding, the team worked to reach a consensus through team discussion. Consensual Qualitative Research (CQR) recommendations were followed whereby one auditor, a nursing professor with extensive qualitative research experience with cancer patients, checked the work of the team [17]. Content analyses were guided by domains derived from the interview questions, i.e., communication experiences across the cancer continuum.

## Results

**Fig. 1:**
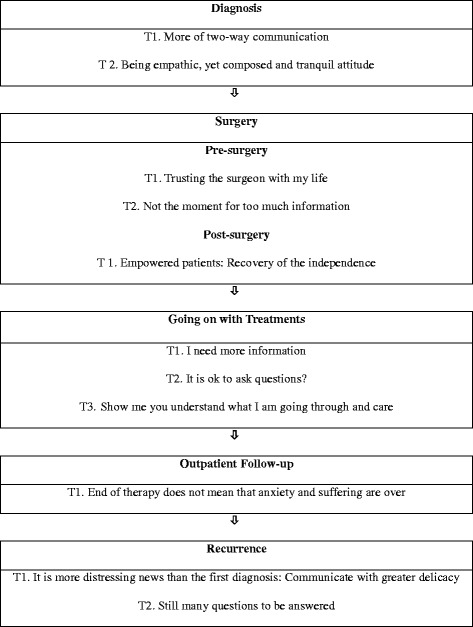
Communications themes across cancer care trajectory

Emerging themes related to communication experiences across the five phases of cancer consultation were shown in Fig. 1.

### Diagnosis

#### Theme 1. More of two-way communication

Feelings of shock, sinking down, surrealism, lack of spirit, and numbness were what patients experienced when they received a cancer diagnosis. These emotional states continued until the end of the consultation, or even after the consultation, making it difficult for patients to participate in the consultation as they were not able to understand or remember explanations or ask questions. As patients are deeply shaken by the diagnosis, they have difficulty understanding or accepting explanations provided to them in a consultation. As such, patients reported that they need to be allowed a sufficient amount of time during the diagnosis as they have to deal with the emotional turmoil associated with receiving a cancer diagnosis. They also reported that doctors need to consider the emotional state of the patient and that it might be helpful to have a moment of silence or give the opportunity to ask questions.

Patients often did not remember the specific content of the consultation and that writing down a memo or drawing was helpful. At this stage, it is more important to explain in a way that patients can understand rather than in a detailed way.

One-way communication is not advisable as it makes patients feel being sentenced, weak, and inhumanely treated. Categorical tones or expressions such as’99.9 % accurate’ or euphemistic expressions of cancer such as ‘a bad illness’ were negatively perceived.

#### Theme 2. Being empathic, yet composed and tranquil attitude

At this phase, non-verbal aspects of communication seemed to be more critical than in any other phase. Patients reported that doctors’ composed and tranquil attitude helped them to deal with their emotions, especially anxiety. Looks of surprise, embarrassment, or regretful looks might be consoling to some patients or in some circumstances, but mostly that wasn’t helpful. Patients wanted a sincere and emphatic attitude from the doctor. They perceived that an eye contact and giving a moment of silence might be ways of expressing the physician’s empathy.

Patients also reported that while a doctor’s composed manner was helpful for them to deal with their emotions, they also want to be treated as a person who just received a cancer diagnosis and not just one of many patients. Patients appreciated doctors’ expressions of empathy and consolation and while some patients wanted these expressed in a straightforward manner, the majority of patients felt that having them expressed in a non-verbal way was sufficient. Patients perceived acts of reassurance and encouragement from doctors about the treatment process as empathic and consolatory.

### Surgery

#### Pre-surgery

##### Theme 1. Trusting the surgeon with my life

As seen in patients’ responses (“*I count everything really on surgeon, dead or alive*”*)*, patients perceived the surgeon as the one who decides whether they live or die, they feel that they cannot do anything but follow him/her completely. As patients experienced concerns about the surgery, doctor’s confidence and reassuring and encouraging words are comforting to patients. In cases where a solid trust was formed between the surgeon and patient, patients willingly followed the surgeon, but most of them felt that they had to follow out of psychological atrophy, desperation, or helplessness. In fact, patients often followed without a sufficient understanding about the treatment and some of them regretted not taking a more active part in the treatment decision making process.

##### Theme 2. Not the moment for too much information

As patients tended to completely depend on the surgeon at this stage and felt that they could not make use of very detailed information. The most pressing concern for patients at this time was about when the surgery would take place and they felt extremely anxious about the delay, even for a day. They seemed to not care so much about receiving detailed information about the surgery or treatments. For instance, patients did not feel helpful explanations focused too much on side effects. However, as individual preferences may vary, it would be better to ask patients’ preferences regarding how much information they want to receive beforehand.

#### Post-surgery

##### Theme 1. Empowered patients: Recovery of the independence

Patients were relieved when doctors gave a clear update that the surgery went well. They finally felt like they were an active agent of treatment and they began to look for what role they could play in their treatment. Patients showed different attitudes from when they made decisions about surgery and they felt that they could make choices independently. Patients’ opinions should be taken into account in decision making about the chemotherapy.

*‘I finally began from now to think about how I am going to receive treatments. I became very active now…”*

*“Now that I’ve received surgery, there are things I want to know more about…”*

*“From now on, it is my problem…”*

It also seems that the expression of empathy and comfort over the loss of patients’ breasts is helpful at this stage. It might be that patients do not show much concern over the loss of their breasts but later it can become an important issue. Giving information about breast reconstructive surgery or the care shown in disinfection was perceived by patients as expressions of empathy and comfort from doctors.

### Going on with treatments

#### Theme1. I need more information

After surgery, most patients receive chemotherapy or radiation therapy and they have both expectations and fears about new doctors and treatments.

Patients perceived that the consultation time was far shorter compared their expectations or needs. They reported that it depended on doctors, but most of them provided information about whether or not chemotherapy was possible and when they were going to start treatment. Patients felt as if there was an atmosphere of “*Don’t ask*”. Patients had supporting staff, like nurses, provide explanations, but this wasn’t a sufficient substitute for that of doctors and the dissatisfaction felt in the consultation with doctors remained. However, patients made use of this information to fill the lack of information.

As patients progressed with chemotherapy treatment and outpatient follow-up, they felt greater anxiety after regular tests, such as blood tests, and wanted detailed explanations about test results. Despite individual differences, most patients felt very dissatisfied when they thought they were not given enough explanation about test results. When doctors did not provide sufficient explanation, they somewhat felt that their rights were violated and doctors were not providing them with sufficient information.

*“I thought the doctor kept information about my results to him/herself. It is like I have to follow him/her blindly and come and go as he/she commands”*

Moreover, information that doctors think is important is not always the same as what patients think is important. In most cases, patients want to hear concrete and exact numbers such as leukocyte counts. Even if the doctor explained the count/numbers are ok, patients were not satisfied with this, and what they wanted to hear was an overall explanation about imaging tests (e.g., the condition of all organs).

#### Theme 2. It is OK to ask questions

Patients communicate actively with their doctor compared to other phases of cancer treatment and have many questions. For instance, they wanted to know about things they could do on their own (e.g., diet, exercise) while on chemotherapy.

*‘I received chemotherapy for the first time, and now I feel I can afford to ask questions… there are many things I wanted to ask…”*

*“For example, principles of the chemotherapy… how to conduct everyday life.. I am very anxious about that…”*

It is important not to dismiss patients’ questions because they may feel hurt and may not ask questions again in the future. A patient reported that after she saw other patients ask questions, but the doctor did not answer, she could not ask questions. Patients also reported a time pressure and somewhat conscious of the limitation of consultation time.

“*Patients have a kind of obsessive worry that they cannot take too much time from doctors, thinking that doctors are always busy and they cannot spend much time on them”*

*“Doctor asks you if you have questions, but it feels like there is no time to ask.. Other people are waiting outside…”*

Therefore, it is important to afford patients at least a minimum opportunity for asking questions and attentively listening to questions.

*“Patients are in a state of extreme perplexity and anxiety. When they ask questions, the doctor does not listen to them and only says what the doctor has to say… Above all, I want doctors to listen”*

*‘”You know when doctors attentively listen to you.. from their answers to my questions or their answers to my questions sufficiently explain in a way that I can understand.. and they answer in a reassuring way without being blunt”*

#### Theme 3. Show me you understand what I am going through and care!

Patients experienced despair and fear when side effects occurred, even if they were expected. When this happens, what patients wanted was reassurance and active concern from their doctor rather than a detailed explanation. Non-verbal aspects of communication were important and expressions that showed reassurance and that doctors cared were important to patients.

*“When I told the doctor that I was suffering to death from vomiting, the doctor said in a genuinely surprised way, ‘It is very regretful. I did not expect you to vomit so much. It felt like the doctor really cared”*

*“There is a difference between saying that “You don’t need to be anxious that might happen” and saying bluntly ‘that’s the way it is”*

When patients cannot tell their complaints to their doctors, they do not report side effects to them and they may control medications at their discretion or even stop taking their medication altogether to control side effects.

It is also important to show an understanding of the patients’ point of view that the chemotherapy is distressing. Patients reported that words of praise and encouragement from doctors over the course of their chemotherapy were helpful and can motivate their compliance with the doctor’s orders.

*‘Since you’ve dealt with surgery well, you can also do well with chemotherapy.” The doctor praised me”*

*“Words of kindness mean so much.. Information about centimeters of the tumor or drugs is important… but when the doctor tells me without delay ‘you are holding up great with the chemotherapy’, recognizing and commending my efforts, that motivates me to do better”*

### Outpatient follow-up after the end of treatment

#### Theme 1. End of therapy does not mean that the anxiety and suffering are over

As patients are familiar with their doctors, consultations at this stage usually took place in a more comfortable atmosphere. However, patients continue to feel anxiety and suffering, even after treatment. Patients reported that they needed detailed explanation and active reassurance and encouragement as ever.

*“Hospital is where you get surgery, chemotherapy, and radiation therapy. When you finish with those treatments, it is really over. But from my experience, after the hospital, with all the treatments, that is when the problems start. I expected to be thrilled about finishing the chemotherapy.. but anxiety about recurrence set in…”*

*“I went to the consultation after having been anxious about test results for a whole week. I mean, for an entire week.. People said that the days of cancer patients are numbered you get tests and are troubled. When they say it is ok you are relieved and yet you get tests again and again, you don’t sleep. I really feel that my days are numbered.*

### Recurrence

#### Theme 1. It is more distressing news than the first diagnosis. Communicate with greater delicacy

In our study, one patient had a recurrence. The patient reported that she was not as shocked as the time of the first diagnosis, but that the situation felt more desperate. Thus, creating an adequate environment to communicate that the cancer had recurred is as important as the time of the first diagnosis.

#### Theme 2. Still many questions to be answered

Even though patients already went through treatments, they have many questions to be answered. They want to know about various treatments and wish to make a choice by themselves, contrary to the phase of the first diagnosis. Also, as metastasis means that cancer progresses, they want to know about the prognosis, but not in terms of how much time remains like the death sentence. They want to hear about the prognosis in a more positive tone if they get treatments.

## Discussion

This study explored experiences of breast cancer patients in cancer consultations and analyzed communication problems and needs of consultations across various phases of the cancer trajectory.

From accounts of patients, it seems that two aspects of cancer need to be addressed in a cancer consultation: the medical aspect and the human aspect of cancer [18]. Interviews revealed that patients perceived cancer also as ‘a disease of the mind’, underlining the notion that cancer care providers should provide appropriate care for the psychological dimensions of the cancer experience in a consultation. Emotional impact of cancer appeared particularly salient in the period of diagnosis, recurrence, and advanced stages of the disease. However, from patients’ perspectives, clinicians’ skills with particular aspects of the consultation, such as “breaking bad news”, tend to be lacking [19]. Patients from our study reported that composure and verbal or non-verbal expressions of empathy from physicians were helpful in dealing with emotions provoked by the cancer diagnosis. Moreover, as suggested by Thorne et al., given the fact that patients are shaken by the diagnosis, cancer care providers should carefully consider the quantity and quality of information given to patients in a consultation and be responsive to patients’ needs and preferences at this stage of heightened emotionality [15, 20]. Recurrence and the transition to advanced stages of the disease constitute another critical moment in which emotional aspects of communication assume significant importance. As patients struggle with uncertainties over the course of the disease, communication at this stage might benefit in helping patients to sustain ‘a thread of optimism’ in the face of uncertainty. Honest communication, balanced with hope, was what patients were asking for [15].

In a related topic, it is necessary to guarantee sufficient consultation time to address the psychological needs of patients in a cancer consultation. However, the reality in most oncology care settings is far from ideal. It seems that patients are well-aware of the limits imposed by short consultation times. While efforts to ensure appropriate consultation time are needed, in the current situation, the effective management of limited time is of considerable importance. One way to achieve that is to give particular attention to non-verbal aspects of the cancer consultation. Non-verbal aspects of communication mattered, and what mattered to patients was not so much ‘what doctors said’, but ‘how they said it’. Non-verbal communication also helped to increase trust towards doctors [21]. Related to this, Thorne et al. found that cancer patients perceived time to be a precious resource in the consultations with care providers and one way of “buffering time challenges” (p. 503) was to use verbal or non-verbal expressions of compassion [22]. Another way of making the most of the limited consultation time is to improve patients’ communication competency in a cancer consultation. Patients need to learn to prioritize communication issues and learn skills to systematically explain their problems. One example of a learning tool is the online program developed by Porter and colleagues in which patients were taught how to communicate their emotions to cancer care providers [23].

Moreover, patients’ psychological state and communication needs evolved according to the context of treatment. As the context of treatment varies, so does what patients perceived important and what they want from doctor-patient communications. Our findings also suggest that as patients progress further in the course of cancer care trajectory, they seemed to be more empowered, playing a leading role in their course of care. Specifically, in the phases of diagnosis and pre-surgery, patients took a passive stance and tended to count on their physicians completely. Thorne et al. recommends that clinicians assist patients’ decision making process with their expert guidance, as patients have to make critical decisions regarding treatment at these early stages [15]. After surgery, patients assume a more active stance, taking more initiatives and asking more questions. As they progress through chemotherapy treatments, patients want more active communication and the need for questioning increases. Thus, it is important for them to be ensured that they will have opportunities to ask questions in the consultation.

Allowing patients to ask questions in a cancer consultation addresses patients’ need for information, which is important for cancer care in a number of ways. First of all, information exchange is a critical communication function throughout the course of the cancer care and will help foster the relationship [24]. Furthermore, when patients experience functional impairments, but have limited information because it was not sufficiently addressed in a cancer consultation, they will interpret these impairments in their own way, possibly leading to increased distress and maladaptive coping [25]. Information given should be comprehensible as it is associated with reduced fear of cancer progression [26]. In addition, in the study by Germeni and Shulz [27], cancer patients showed both information seeking and avoidance patterns and the motivating themes behind these two patterns varied from the initial diagnosis to the treatment phase, suggesting a need for phase-specific information provision.

Additionally, Thorne et al. [15] suggested that as patients conclude treatment, they were confronted with new anxieties associated with symptom management and vigilance over their state of health, in particular recurrence. Effective communication at this stage should pay attention to assisting patients in their attempt to assume a more active role and take on more responsibility in their cancer care. Furthermore, as patients continue to feel anxiety and suffering even after treatments are completed, as observed in our study, caring for these emotional issues with empathy will be necessary as these emotional issues have their cost. For instance, fear of recurrence was associated with higher health costs and lower monitoring rates [28].

Van Dalen suggests that the effectiveness of communication will increase depending on the degree in which physicians attempt to understand the patient’s perspective, which includes the patient’s context [25]. The specific phase in which patients are in, with specific challenges and needs in the cancer trajectory, might constitute such context and effective communication should involve addressing these challenges and needs. This study is one of only a few such efforts that explored the communication experience of breast cancer patients, from their own perspective, throughout the continuum of cancer care. Current findings can provide evidence-based recommendations for communications with cancer patients and take into consideration the phase-specific communication needs of patients.

However, the study findings should be considered within the limitations. The sample size was relatively small and the participants were breast cancer patients who are relatively young and participating in a meditation program at a breast cancer clinic. Thus, the generalizability of the findings is limited and potential selection bias should be considered in the interpretation of the findings.

Moreover, as communication needs and preferences might vary also in terms of the patients’ personal characteristics, such as age and gender, further studies should be carried out with other types of populations. For instance, while older patients want information regarding the disease and treatments like younger patients, they showed less interest in the details [29].

## Conclusions

To conclude, current findings suggest that the efficacy of communication varies depending on which phase patients are in and that effective communication should be tailored to the evolving needs and preferences of breast cancer patients. Also, as patients perceived that the consultation did not adequately address their need for information related to their care or their emotional issues associated with the cancer experience, it is important to address their needs by paying particular attention to non-verbal aspects of communication that convey empathy and respect toward patients, as well as allowing patients to ask questions.

## Additional file

Additional file 1: Semi-structured interview questions. Semi-structured interview questions used in the study. (DOCX 24 kb)

## Abbreviations

CQR: Consensual qualitative research
CTx: Chemotherapy
Op: Op: operation
RTx: Radiation therapy
TX: Treatment
