# Supplementary material for: Tailoring communications to the evolving needs of patients throughout the cancer care trajectory: a qualitative exploration with breast cancer patients
Source: BMC Womens Health. 2016 Oct 18;16:65. doi: 10.1186/s12905-016-0347-x (PMC5069888; doi:10.1186/s12905-016-0347-x)
Supplement: Additional file 1: — Semi-structured interview questions. Semi-structured interview questions used in the study. (DOCX 24 kb) [file 12905_2016_347_MOESM1_ESM.docx]

| **Semi-Structured Interview Questions**   1. 1. Please tell us about your communication experience from when you were in consultations with doctors as a cancer patient 2. 2. Could you tell us more about the most impressive or distressing consultation you’ve experienced? 3. 3. Tell us about your positive experience while you were in a consultation with doctors. 4. 4. Tell us about uncomfortable or distressing experience during a consultation with doctors.   4-1. Were there things that doctors could do to make things better for you?   1. 5. Now, let us talk about your consultation experience at each stage of your cancer care.   5-1. Tell us about the consultation at the first time when you’ve been diagnosed with cancer.  5-2. Tell us about the consultation when you have decided to get cancer treatment (surgery, chemotherapy, radiation therapy, immune therapy, etc.).  5-3. (If you experience side effects of treatment), tell us about the consultation experience when you discussed side effects of the treatment.  5-4. Tell us about the consultation when you heard about treatment courses or results, either during treatment or afterwards.  5-5. If you have experienced recurrence or metastasis, tell us about the consultation at that time.  - Additional questions regarding the consultation experience at each stage of cancer experience  How long did you have consultation with doctors?   1. 1) What do you remember about the atmosphere of the consultation room at that time? 2. 2) Was there someone with you at the time of consultation? If so, was it helpful for you? 3. 3) What call you tell us about your doctors’ eye-contact or posture? What do you remember about the tone of their voices or the ways the doctors talked to you? 4. 4) How did your doctor explain about the illness or treatment? Did doctor explain the medical terms in a way that you could understand?   5) Did the doctor provide you with a detailed explanation about your illness or treatment? To what extent were you able to understand the information provided?  6) How did the doctor explain side effects of the treatment? What did you think about these explanations?  7) How did you decide about your treatment? What did you think about your decision at the time?  8) How did the doctor explain some serious matters, such as the prognosis or survival rates? How did you feel about the explanations?  9) How much questions did you ask from the doctors and what kind of questions were they?  10) Did the consultation with doctors help you to pursue with your treatment? If so, tell us what words the doctor told you. If it did not help you, what were the words that the doctor told you?  11) How well did your doctor listen to you?  12) How well did your doctor show empathy to your suffering? |
| --- |
